# Evaluation of the Performance of Algorithm-Based Methods for Subjective Refraction

**DOI:** 10.3390/jcm9103144

**Published:** 2020-09-29

**Authors:** Abinaya Priya Venkataraman, Delila Sirak, Rune Brautaset, Alberto Dominguez-Vicent

**Affiliations:** Section of Eye and Vision, Department of Clinical Neuroscience, Karolinska Institute, 17177 Stockholm, Sweden; delila.sirak@stud.ki.se (D.S.); rune.brautaset@ki.se (R.B.); alberto.dominguez.vicent@ki.se (A.D.-V.)

**Keywords:** subjective refraction, algorithm, agreement

## Abstract

**Objective:** To evaluate the performance of two subjective refraction measurement algorithms by comparing the refraction values, visual acuity, and the time taken by the algorithms with the standard subjective refraction (SSR). **Methods:** The SSR and two semi-automated algorithm-based subjective refraction (SR1 and SR2) in-built in the Vision-R 800 phoropter were performed in 68 subjects. In SR1 and SR2, the subject’s responses were recorded in the algorithm which continuously modified the spherical and cylindrical component accordingly. The main difference between SR1 and SR2 is the use of an initial fogging step in SR1. **Results:** The average difference and agreement limits intervals in the spherical equivalent between each refraction method were smaller than 0.25 D, and 2.00 D, respectively. For the cylindrical components, the average difference was almost zero and the agreement limits interval was less than 0.50 D. The visual acuities were not significantly different among the methods. The times taken for SR1 and SR2 were significantly shorter, and SR2 was on average was three times faster than SSR. **Conclusions:** The refraction values and the visual acuity obtained with the standard subjective refraction and algorithm-based methods were similar on average. The algorithm-based methods were significantly faster than the standard method.

## 1. Introduction

Our vision is determined by both optical and neural factors, and subjective refraction considers these factors to determine the optimal optical correction. The objective refraction only considers the optical factors. Due to this, the subjective refraction which takes into account both optical and neural factors is used to determine the final optical correction. The subjective refraction is often described as the gold-standard method though the procedure varies widely. The methods used to perform subjective refraction mainly vary in terms of steps used to determine best sphere, usage of duochrome, the technique used to determine cylinder and binocular balance. It has been recommended that subjective refraction can be used as a gold standard when new refractive procedures are assessed if the procedure for subjective refraction is fully described [1]. 

Apart from the neural factors, the difference between the objective and subjective refraction could also be explained by instrument related factors. Comparing the objective and subjective refraction, the accuracy of objective refraction measurement is shown to be good with open-field view autorefractometers [2,3,4]. Instrument myopia can be induced with closed-field view of the autorefractometers and whether there is a fogging step used during autorefractometer measurement [5,6,7]. Image quality metrics based on the wavefront data is also widely used to optimize the objective refraction and predict the subjective image quality [8,9,10,11].

Subjective refraction procedures are time consuming due to the various steps involved and the time taken by the subject to respond. Previous studies that compared the manual subjective refraction procedure with algorithm based automated procedures reported that the latter method was significantly faster [11,12]. In addition to this, the outcome of the manual subjective refraction procedure can be influenced by the examiner’s experience and the criteria set for determining the end point [1,13]. The automation of subjective refraction can optimize both the time spent on refraction and reduce examiner bias. The automated algorithms require the subjective response to be inputted during refraction and based on the response the algorithm decides the subsequent steps [12,14]. A previous study demonstrated a significant reduction in the time spent on subjective refraction with a semi-automatic algorithm from a binocular aberrometer incorporated with a phoropter [12]. 

There are different automated algorithms available which are incorporated in phoropters. The Vision-R 800 phoropter (Essilor, Paris, France) includes a set of subjective refraction routines where the subjective responses are used by the algorithm to adjust the sphere and cylinder. This phoropter has a possibility to change the power continuously in 0.01 D steps, and also simultaneously change the sphere and cylindrical components when the algorithm-based methods are used.

A combination of the automated algorithm and continuous power change can help to reduce the time spent on refraction as well as provide more precise refraction. With the new algorithms, it is crucial to evaluate the efficiency of the performance. In this study, we evaluated two different algorithms available in Vision-R 800 phoropter. The refraction values, final visual acuity, and the time taken by the algorithms were compared with the standard subjective refraction. We also evaluated the agreement between the refraction values obtained with the objective and the different subjective refraction methods.

## 2. Material and Methods

### 2.1. Subjects

This prospective study included 68 subjects (54 females) in the age range of 18 to 40 years. The participants were recruited in the Optometry clinic of the institution. The study followed the tenets of the Declaration of Helsinki, and all participants provided written informed consent and the study was approved by the regional ethics committee. The inclusion criteria to participate in this study were no current use of Ortokeratology lenses, no history of ocular disease including binocular vision disorders or prior ocular surgery, intraocular pressure below 21 mmHg, no pregnancy or lactation currently, and no use of any systemic or ocular medication that could have any impact on the refraction

### 2.2. Materials

The objective refraction (OR) was measured using the Waveanalyzer 700 (Essilor, France). This instrument uses a Hartmann-Shack sensor to measure the wavefront and calculate the objective refraction. The refraction is measured monocularly under closed-view condition. The subjects were instructed to fixate at the instrument’s internal fixation target during the measurements. 

All the subjective refraction evaluations were performed in the Vision-R 800 phoropter. This phoropter contains liquid lenses and allows smooth power change during subjective refraction. With this phoropter both the standard subjective refraction procedure and semi-automated procedures (referred to as smart method) that use in-built algorithms were used to determine the subjective refraction. 

### 2.3. Procedure

In total, three different binocular subjective refraction measurements were performed on each participant: subjective refraction performed without any automated algorithm, which will be referred to henceforth as the standard subjective refraction (SSR) method and two different smart subjective refraction (SR1 and SR2) methods. The OR value was used as the starting point for all three methods. The spherical and cylindrical refinements were done in ±0.25 D steps for SSR and in ±0.10 D steps for SR1 and SR2. All the subjective refraction procedures were performed in the same Vision-R 800 phoropter and the digital screen placed at 5 m. Visual acuity with the OR value was recorded initially.

For the SSR, the method for binocular subjective refraction described in Borish’s clinical refraction [15] was used. After an initial binocular fogging, monocular defogging was performed considering the criteria of maximum plus or minimum minus providing the maximum visual acuity. Then the cylinder was refined with the cross cylinder technique. After the monocular endpoint was determined for both eyes, the binocular balance was checked with fogging and alternate occlusion. The final binocular endpoint was determined by binocular fogging and defogging. In this method, the examiner modified the lenses according to the subject’s response in each step. 

The smart methods, SR1 and SR2, are algorithm based subjective refraction procedures. Both methods use the subjective response on visual quality in the algorithm and simultaneously modify the spherical and cylindrical component to reach the endpoint of the subjective refraction. In SR1, after an initial defogging up to the maximum visual acuity, a duochrome test was used for monocular spherical refinement and cross cylinder was used for cylindrical refinement. This was followed by a polarization test for binocular balancing and a binocular duochrome test for the binocular endpoint. SR2 is similar to SR1, except that there was no initial fogging of the examined eye and the duochrome test was performed directly from the starting value. In both SR1 and SR2, the examiner entered the subject’s response and the algorithm automatically modified the lenses accordingly.

The measurements on each subject were conducted on two consecutive days. On one day, the SSR was performed and the two smart tests (SR1 and SR2) were performed on the other day with a five-minute break in between. The order of these was chosen randomly. The OR measurement was carried out on day 1 before the subjective refraction procedures. The examiner followed the steps and criteria for all the subjective refraction procedures in order to avoid bias. All the measurements were performed in the same room with the same illumination. The time taken for each subjective refraction procedure, the final refraction values, and the visual acuity were recorded. 

### 2.4. Statistical Analysis

For further analysis purposes, the objective and subjective refractions were converted into power-vector notation [16] as the following,

$$M=S+\frac{C}{2}$$

$$J0=-\frac{C}{2}\cdot \cos2\cdot \alpha$$

$$J45=-\frac{C}{2}\cdot \sin2\cdot \alpha$$

*S* represents the spherical power, *C* represents the negative value of the cylindrical power, and α represents the cylinder axis. *M*, *J*0 and *J*45 represent the spherical equivalent and the two cylindrical vectorial component. Only the values from the right eyes were included in the analysis. An ANOVA was also performed to find out whether there were differences among the refraction values, visual acuity with each refraction, and the time taken for the three different subjective refraction procedures. The post-hoc comparison was performed with the Tukey test. The statistical significance limit was set to a *p*-value < 0.05. A Bland-Altman analysis was used to assess the agreement among the refraction values [17] between the SSR and the other subjective methods. The agreement between the two smart methods (SR1 and SR2) was also performed. All analyses were performed in Matlab.

The sample size required was calculated to be 60 subjects. This was based on the calculations for the sample size for agreement studies for the confidence interval to be 0.05 D for the estimated limits of agreement [18].

## 3. Results

The average age of the subjects was 25.1 ± 4.1 years. The refraction values from each method, visual acuity, and time taken are summarized in Table 1. The differences between the different methods for *M*, *J*0, *J*45 and visual acuity were not statistically significant (*p* value: 0.638, 0.827, 0.813, and 0.187 respectively). The times taken for each subjective refraction methods were significantly different (*p* < 0.05). The times taken for both SR1 and SR2 were significantly less than that of SSR. On average, the SR2 was about three times faster than the SSR.

**Figure 1:**
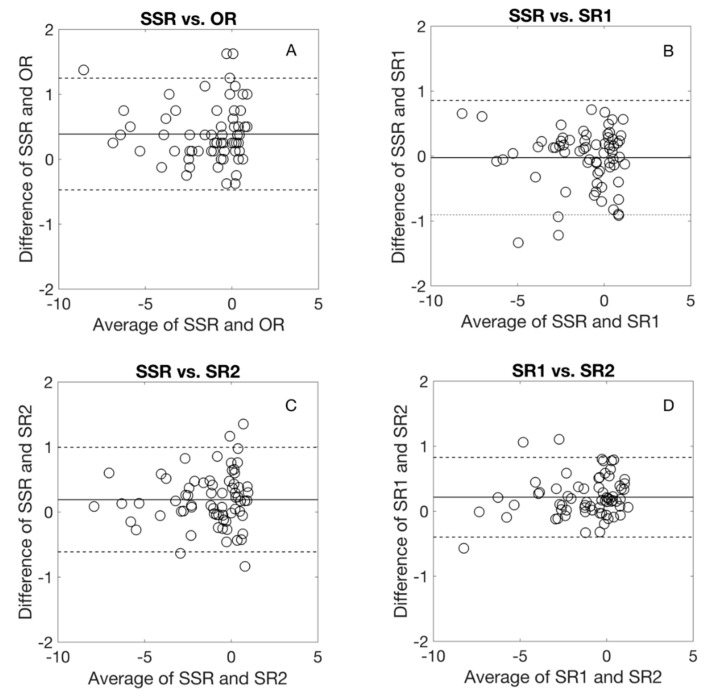
Bland-Altmann comparison for spherical equivalent among different refraction procedures. The middle line represents the average difference, the dashed lines represent 95% limits of agreement. OR: Objective refraction, SSR: Standard subjective refraction, SR1 and SR2: the two algorithm-based subjective refraction procedures.

Figure 1 shows the Bland-Altman comparison of *M* with the different refraction methods. On average, the *M* was more positive with SSR by 0.4 and 0.2 D compared to OR and SR2, respectively. The difference between SSR and SR1 was almost zero and between SR1 and SR2 was 0.2 D. The agreement limit interval between the SSR and the other refraction methods was about 1.75 D. For SR1 and SR2, the agreement limit interval was about 1.25 D.

**Figure 2:**
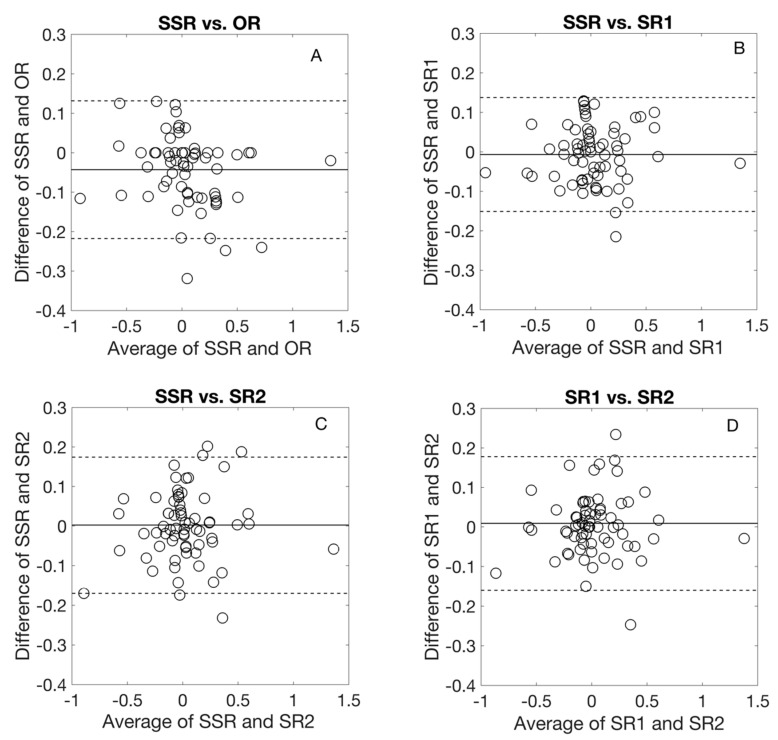
Bland-Altmann comparison for cylindrical component, *J*0 among different refraction procedures. The middle line represents the average difference, the dashed lines represent 95% limits of agreement. OR: Objective refraction, SSR: Standard subjective refraction, SR1 and SR2: the two algorithm-based subjective refraction procedures.

**Figure 3:**
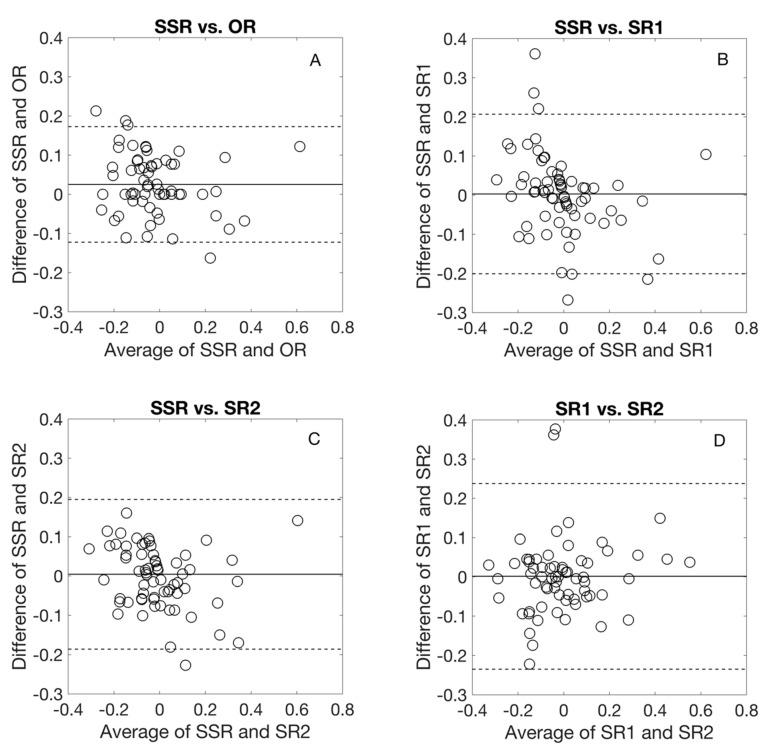
Bland-Altmann comparison for cylindrical component, *J*45 among different refraction procedures. The middle line represents the average difference, the dashed lines represent 95% limits of agreement. OR: Objective refraction, SSR: Standard subjective refraction, SR1 and SR2: the two algorithm-based subjective refraction procedures.

Figure 2 and Figure 3 show the Bland–Altman comparison of *J*0 and *J*45 with the different refraction methods. For both *J*0 and *J*45, the average difference was almost zero and the agreement limit interval was less than 0.50 D for all comparisons.

## 4. Discussion

We evaluated the performance of two algorithm-based methods by comparing the outcome with the manual standard method. The algorithm-based methods used in this study differ mainly in terms of the initial fogging steps. On average, the algorithm-based methods provided a similar outcome compared to the standard method, both in terms of refraction value and visual acuity. The average differences in the spherical equivalent component obtained with the different refraction methods was less than 0.50 D. The agreement limit interval for these comparisons was less than 1.75 D. For the cylindrical components, the average difference was close to zero and even the limits of agreement intervals were less than 0.50 D. A notable difference was seen in the time taken for the algorithm-based methods which were significantly faster than the standard method. These results provide information on the efficacy of these algorithm-based methods in determining subjective refraction.

The SSR resulted in less negative M than OR, which is expected as the instrument used for OR measurement was a monocular closed view autorefractometer which can induce proximal myopia [6,19,20,21]. Comparing SR1 with the standard method (SSR), the average difference in the M was almost zero. In SR1, the initial fogging and defogging steps (SR1) was used which is similar to that of SSR. A previous study comparing standard subjective refraction with another algorithm-based procedure which also used the fogging steps showed similar average spherical equivalent values [12]. When the initial fogging steps are skipped (SR2), the standard method provided on average 0.20 D less myopic refraction. Similarly, SR1 which included initial fogging provided less myopic refraction compared to SR2. This could be the consequence of using the OR value as the starting point without subsequent fogging in SR2. The first duochrome test was performed before the cross-cylinder refinement in both algorithm-based methods. It has been reported that the duochrome test performed either before or after cylindrical refinement results in similar spherical outcomes [22]. The SSR method did not use the duochrome test for estimating the spherical end point.

Although the average difference in the spherical equivalent was less than 0.50 D in all of the comparisons performed in this study, this parameter alone does not provide information about the clinical relevance. The limits of agreement interval has to be considered during the interpretation of the results as this is the interval within which 95% of the differences between the measurements can lie. The limits of agreement intervals were about 1.50 to 1.75 D for the comparison of the spherical equivalent between the standard method and the algorithm-based methods (SR1 and SR2). This interval is wide suggesting that the algorithm-based methods can provide more myopic or less myopic refraction. Comparing SSR and SR1, the average difference is zero but SSR can measure up to 0.8 D less myopic or 0.9 D more myopic. For SSR and SR2 comparison, the average difference is 0.2 D but SSR can measure up to 1.0 D less myopic or 0.6 D more myopic. From a clinical point of view, these differences are significant. Based on these results, it can be suggested that when the clinician uses the algorithm-based methods, further refinements might be needed to determine the end point of subjective refraction. The algorithm used in this study has not been evaluated previously. However, the limits of agreement intervals obtained in this study are in the same magnitude as in the previous reports on evaluations of the different subjective procedures (with and without algorithm) and its comparison with the objective procedures [12,23,24].

The algorithm-based methods alter the spherical and cylindrical component simultaneously when required, whereas in SSR this is not done simultaneously. Apart from the other differences between the methods, the continuous and simultaneous change of lenses can also influence the time taken. The present study included young adults (18–40 years) with no other ocular disorders. Both SSR and SR1 include an initial fogging and defogging step which plays a role in accommodation control. The performance with the algorithm-based methods could be different in children and presbyopes. The major advantage of using algorithm-based methods is the shorter time taken compared to the standard method while resulting in similar visual acuity. The shorter time spent on refraction allows more time for further examination of the visual system in clinical practice. The results on the limits of agreement interval suggest that a final spherical refinement step could be added along with the duochrome test. Even with this, the procedure might still be faster compared to the standard subjective refraction methods.

For the cylindrical components *J*0 and *J*45, the average difference was close to zero and even the limits of agreement intervals were less than 0.50 D. In this study, the maximum astigmatism included was about 3.0 D. The results might be different in eyes with higher astigmatism or irregular corneas. The algorithms might result in precise outcomes even in such cases as when the cylindrical adjustment is performed simultaneously throughout the procedure. In addition to this, the phoropter allows for further smaller steps (as low as 0.01 D), which can result in better cylindrical refinement in these eyes. The efficacy of refraction performed with smaller increments in power and evaluation of performance in different age groups, high astigmatism, and irregular corneas needs to be evaluated.

In conclusion, the algorithm-based methods evaluated provided similar results on average compared to the standard subjective refraction in terms of the refraction values and visual acuity. The algorithm-based methods are also significantly faster than the standard method.

## Figures and Tables

**Table 1:** Descriptive statistics from the different refraction procedures.

	OR	SSR	SR1	SR2
*M*	−1.51 ± 2.09	−1.12 ± 2.07	−1.07 ± 2.12	−1.30 ± 2.06
*J*0	0.07 ± 0.33	0.03 ± 0.31	0.03 ± 0.31	0.03 ± 0.31
*J*45	−0.03 ± 0.16	−0.01 ± 0.15	−0.01 ± 0.18	−0.02 ± 0.18
VA (logMAR)	−0.18 ± 0.15	−0.18 ± 0.10	−0.12 ± 0.20	−0.18 ± 0.11
Time (minutes)	NA	9.52 ± 1.59	5.41 ± 0.98	3.14 ± 0.60

OR: Objective refraction; SSR: Standard subjective refraction; SR1 and SR2: Two algorithm-based subjective refraction procedures; *M*: Spherical equivalent. Expressed in Dioptres. *J*0 and *J*45: Cylindrical vectorial components. Expressed in Dioptres. VA: Visual acuity.
